# Electrical Heating Performance of Electro-Conductive Para-aramid Knit Manufactured by Dip-Coating in a Graphene/Waterborne Polyurethane Composite

**DOI:** 10.1038/s41598-018-37455-0

**Published:** 2019-02-06

**Authors:** Hyelim Kim, Sunhee Lee, Hanseong Kim

**Affiliations:** 1 Research Institute of Convergence Design, Dong-A University, Busan, 49315 Republic of Korea; 2 Dept. Fashion Design, Dong-A University, Busan, 49315 Republic of Korea; 3 Dept. Organic Material Science and Engineering, Pusan National University, Busan, 46241 Republic of Korea

## Abstract

An electro-conductive para-aramid knit was manufactured by a dip-coating in a graphene/waterborne polyurethane(WPU) composite for confirming to use as a fabric heating element applicable to a protective clothing requiring durability. The para-aramid knit was dipped in 8 wt% graphene/WPU composite solution up to five-coat cycles. As a result of electro-conductive textile by number of dip-coating cycles, the electrical, and specifically electrical heating performances were increased number of cycles from one to five. The sample with the best electrical and electrical heating performance was the five-coat sample, and to improve those properties it was hot-pressed at 100 °C, 120 °C, 140 °C and 160 °C. After hot pressing, the entire surface of the sample was filled with graphene/WPU composite and indicated smoothly surface, thus the electrical and electrical heating performance was improved than the five-coat sample. The best performance of was indicated hot-pressed at 140 °C, with a surface resistivity and capacitance of 7.5 × 10^4^ Ω/sq and 89.4 pF, respectively. When a voltage of 50 V was applied, the surface temperature reached 54.8 °C. The five-coat sample with hot-pressed at 140 °C could be applied to a heat-resistant para-aramid knit glove with the touch screen of a mobile phone and electric heating performance.

## Introduction

Electronic textiles, or e-textiles, are a class of fabric structures with integrated electronic elements. The incorporation of electrical conductivity in textiles can add a new dimension to their application potential^1^. To improve the electrical conductivity, conductive materials can be used as electro-conductive textiles, such as carbon-based nanomaterials^2,3^ or graphene-based materials^4–11^. The types of materials are mainly films^2,3,11^, yarns, textiles^12,13^, and fabrics^14–20^. They can be used for supercapacitors^4^, sensors such as strain and piezo sensors^5–8^, and electrical heating materials^2,3,9–11^ in wearable textile electronics. The electrical heating fabric is one of the e-textiles which are used to maintain body temperature in extreme environments such as in work clothing, protective clothing, winter sports clothing, and military uniforms, etc., and those products need to light-weight, flexible and durability for activities^2,3,9–11,21^.

Graphene is a new and exciting material that has high electrical conductivity, high thermal conductivity, high flexibility, and good thermal and chemical stability^22,23^. Solution mixing is the most commonly used technique to fabricate polymer composites. Graphene has been extensively used as fillers in composites because of the excellent properties that result. Studies have been conducted on graphene as a filler and polyurethane as a matrix for coatings and adhesives^8,9^. Recently, waterborne polyurethane (WPU) has been extensively used instead of polyurethane (PU) due to its low content of volatile organic compounds (VOCs)^24^. To improve the durability of WPU, the incorporation of small amounts of graphene may enhance the electrical, mechanical, and thermal properties. Also, when the graphene fillers form the percolation threshold in the WPU matrix, the electrical conductive path is created and electrical properties are improved^10,25^.

Dip coating is a simple method for coating graphene onto fabrics or textile fibers^14–20^. This method is capable of producing very thin membranes and is promising for high adhesion, reproducibility, and preparation at a large scale. Chatterjee *et al*.^16^ studied the electrical conductivity of cotton woven and knitted fabrics with different concentrations of graphene oxide (GO) and the number of coating cycles. They used up to 15 coating cycles, which increased the concentration of GO and the electrical conductivity. Silk fabric was repeatedly dipped into a reduced graphene oxide (rGO) mixture^17^. The number of rGO sheets increased with the increasing of coats. Thus, the surface resistivity of silk fabrics with 4, 6, and 9 coats decreased to 62.42, 14.75, and 3.24 K Ω cm^−1^, respectively. Previous studies reported that fabricated electrical heating elements using dip coating with graphene composite were dip-coated on nonwoven fabric^9,19^. Liu *et al*.^9^ reported that the heating performance for the composite fabrics prepared in an aqueous dispersion containing 0.080 wt% rGO indicated a steady-state temperature of 59 °C within 30 min at 30 V. Kongahge *et al*.^19^ was analyzed the thermal behavior of the rGO nonwoven textiles, and the sample of fabric measuring 200 mm × 200 mm with 6 wt% of rGO was heated to an average temperature of 36 °C with 32.5 V and 0.05 A within 10 min.

Hot pressing^26^ is the simplest route to produce multilayer samples. The process involves pressing independent layers at high temperature. When the layers are heated under high pressure, they stick together via mechanical bonds. The samples then become highly dense, and composite is impregnated in the samples. Cataldi *et al*.^27^ studied a healable cotton-graphene/thermoplastic polyurethane nanocomposite conductor for wearable electronics. Folding induced microcracks were applied as defects that affected the properties, and the conductor could be healed easily by hot-pressing to restore the initial electrical conductivity.

Generally, fabrics such as cotton or polyester are mainly used for fabricating electro-conductive textiles due to commercial availability, usefulness, comfort, and low cost^14–19^. These fabrics can be used in general garments, but they are difficult to use when garments are required durability like bending or friction. Para-aramid fabrics are used in the elbows, knees, and hips of stretchable and durable garments, as well as gloves that require heat resistance and cut resistance. Previous research using para-aramid and graphene has been reported to improve the mechanical and electrical properties of a graphene-aramid nanofiber nanocomposite^28^. Wearable electronic textiles manufactured by dip-coating carbon nanotubes on Kevlar to fabricate smart armor were reported that the stab resistance performance and dynamic impact resistance was increased compared with the neat Kevlar, and it can achieve a stable electrical conductivity of ~10^−2^ Sm^−1^.

In the present study, as-fabricated graphene/WPU composite dip-coated para-aramid knit textile which possessed the electrical heating performance was prepared to apply the functional clothing that required flexible, high durability and heat resistance, such as protective clothing, outdoor or leisure sports clothing, etc. Thus, a graphene/WPU composite were dip-coated on the para-aramid knit up to five-coat and then hot-pressed the five-coat sample which indicated the best performance due to enhance the electrical and electrical performance. We also confirmed its applicability to functional clothing requiring durability by establishing optimal conditions. The specific objectives were as follows. First, the 8 wt% graphene/WPU composite solution was prepared, and dip coated on para-aramid knit from one to five times, and then the characteristics of samples by the number of coating cycles. Second, the electro-conductive textile with the best performance was hot-pressed at 100 °C, 120 °C, 140 °C, and 160 °C, and then the characteristic were analyzed according to the hot-press temperature. Finally, the electro-conductive textile was applied to the para-aramid knit glove to confirm its electrical and electrical heating performances as an e-textile.

## Results and Discussion

**Figure 1:**
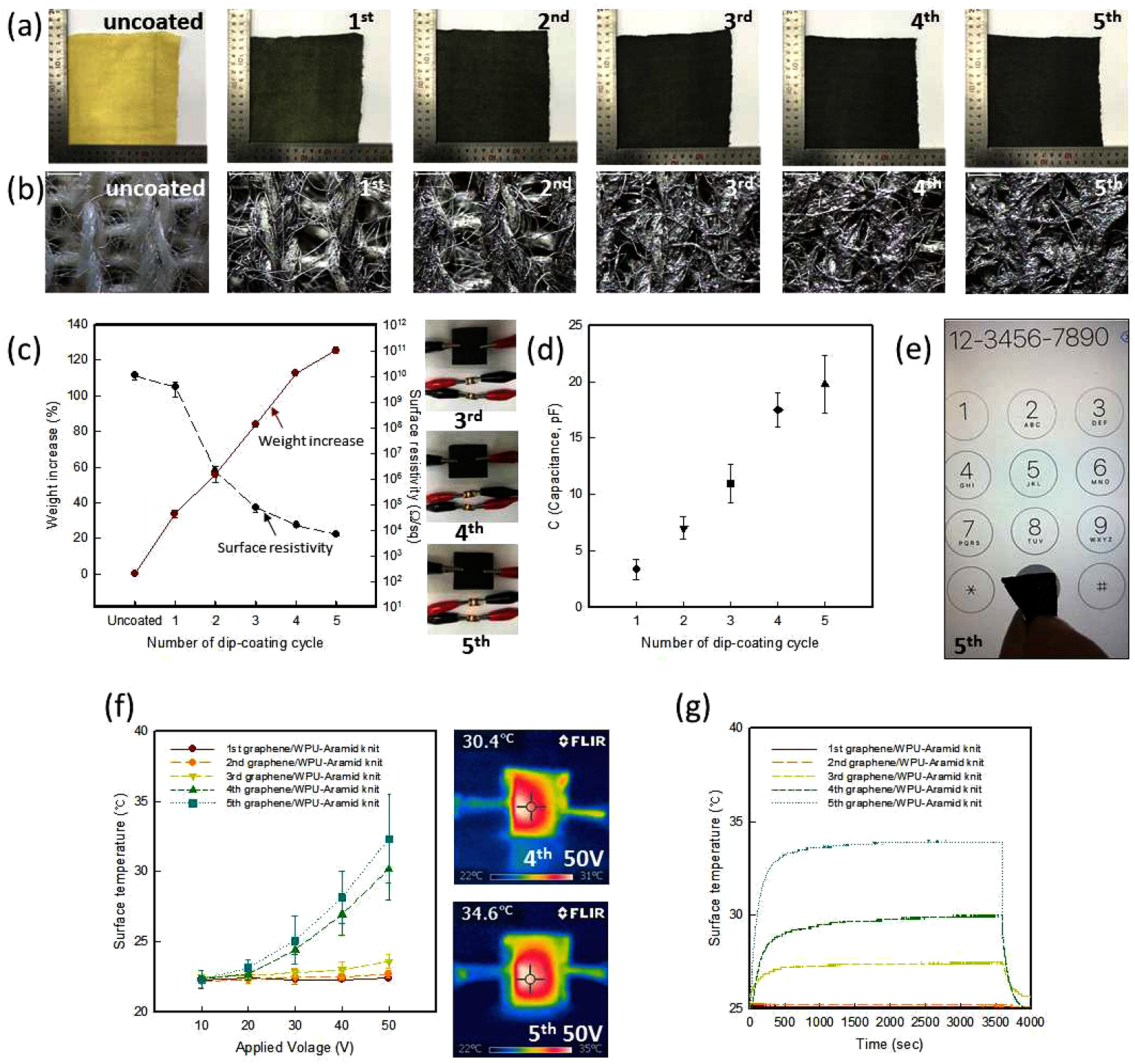
(**a**) Digital images and (**b**) morphology of graphene/WPU dip-coated para-aramid knits, (**c**) Weight increase and surface resistivity of electro-conductive textile (insert image shows using the three, four, and five-coat samples to light up two LEDs) (**d**) Capacitance of dip-coated electro-conductive textiles, (**e**) Using the five-coat samples to touch a screen, (**f**) Electrical heating properties of the electro-conductive textile (insert image indicates IR thermal image of four and five coat samples when applied 50 V), (**g**) Electrical heating behavior of samples by number of coating cycles with applied 50V.

The dip-coated knits were indicated an obvious color change as the dip-coating cycles increase compared with the uncoated para-aramid knit (Fig. 1(a)). The para-aramid knit was dip-coated up to six times as seen in the Fig. S1, but the sample coated six cycles with graphene/WPU composite solution was brittle and some cracks were expressed on the surface. Thus, it confirmed that was not good at formability, this study indicated results of dip-coated samples up to five times. The uncoated knit was yellow, but the color became closer to black with increasing coating cycles. The detailed morphology of the knits is shown in Fig. 1(b). The uncoated knit had a porous area between courses and wales and between fibers. However, with increasing dip-coating cycles, the porous area was packed with the graphene/WPU composite. The surface resistivity of the samples was analyzed in relation to the weight increase with the number of dip-coating cycles (Fig. 1(c)). As the weight increased with the coating cycles, the surface resistivity of the samples decreased. The surface resistivity of uncoated and one-coat knit were 1.1 × 10^10^ ± 3.5 × 10^9^ Ω/sq and 4.0 × 10^9^ ± 2.3 × 10^9^ Ω/sq, respectively. However, when increasing the dip-coating cycles from 2 to 5 times, the surface resistivity decreased from 1.9 × 10^6^ ± 1.2 × 10^6^ Ω/sq to 3.1 × 10^4^ ± 1.5 × 10^3^ Ω/sq, and the weight increase changed from 38.70% to 125.50%. The electrical properties were thus enhanced with increasing amount of graphene added to the knit. It was shown that the three, four, and five-coat samples could be used as an electrical conductor to light two light-emitting diodes (LEDs). Chatterjee. *et al*.^16^ reported that the surface resistivity of cotton woven fabric and knit fabric were 0.26 MΩ/sq and 0.19 MΩ/sq after 15 dipping cycles with a 2.25% GO concentration. This may be due to the higher graphene added on to knitted fabric (3.96%) than that of the woven fabric (3.30%). Cao *et al*.^20^ fabricated a multifunctional silk fabric via graphene oxide repeatedly coating. They reported the four-coat silk sample and eight-coat silk sample could be used as an electrical conductor to light the LED lamps, but the brightness of the LED lamps for the eight-coat silk sample is higher than that of four-coat silk sample. And the surface resistivity of four-coat and eight-coat silk samples indicated about 60 kΩ/cm and 5 kΩ/cm, respectively, it was reported indicating the lower surface resistance of eight-coat silk sample. With more graphene added to the fabric, it was possible to make more connections between the graphene and fiber matrix due to the increased number of conductive pathways.

Most fabrics reduce the conductance of the skin by blocking the current passing from the skin to a touchscreen and reducing the pressure sensitivity. Conductive fabrics for touchscreens must be able to conduct electrical currents from the wearer’s skin. The resistance of a conductive material simply refers to how well the material can conduct a charge from skin to a capacitive touchscreen^29,30^. To investigate the performance of the conductive textile for a touchscreen, the capacitance value of the knits was measured, as illustrated in Fig. 1(d). With increasing number of coats from 1 to 5, the capacitance increased from 3.4 to 19.8 pF. As the capacitance increases at the same conditions of voltage, frequency, contact area, and diameter, the amount of charge that can be accumulated in the knit increases as the number of coats increases. The capacitance of the five-coat sample was the largest. As shown in Fig. 1(e), the five-coat sample was successfully used on a smartphone touchscreen (see Supplementary Video Demonstration). This means that the charges in the five-coat sample were adequate to used touchscreen compared to the samples with one to four coats and could be transfer from a finger to the screen^31^.

For confirming the electrical heating properties of electro-conductive textiles dip-coated with graphene/WPU composite, the surface temperature of samples was shown in Fig. 1(f). The voltage was applied to the samples, and their response was recorded by and infrared camera. As the number of coating cycles increases, the electric heating properties were improved. When the 50 V was applied to samples with one to three coats, the surface temperature indicated 21.8 ± 0.0 °C, 22.0 ± 0.3 °C, and 23.6 ± 0.5 °C, respectively. The surface temperature was increased from one to three coats but it was hardly observed the electrical heating properties because those samples were indicated almost no variation of the surface temperature when the voltage was applied from 10 V to 50 V. When 50 V was applied, the four-coat and five-coat samples reached temperatures of 30.2 ± 4.1 °C and 32.4 ± 3.2 °C, respectively. From the four-coat sample was shown over than 30 °C as applied 50 V and the different was over than 10 °C compare with one to three coats samples. It was related to the surface morphology and surface resistivity shown in Fig. 1(b,c).

As shown in the Fig. 1(b), samples with one to three coats indicated the amount of graphene/WPU composite coated on the para-aramid knit increased with increasing number of coatings, but the samples were not sufficiently coated with the graphene/WPU composite solution and the samples had the voids in the surface, that made poor regularity and connectivity^20,32^. However, samples with four and five coats expressed hardly voids in the surface and filled with graphene/WPU composite and expressed more regularity surface than samples with one to three coats. As seen in the Fig. 1(c), the samples with one to three coats expressed the large variation of the surface resistivity and weight increase, however the samples with four and five coats indicated the small variation of the surface resistivity and weight increase. Therefore, the abundant conductive networks were formed in the knit as the number of coating cycles increases.

The electrical heating performance can be explained by Joule heating, where the electrical power is defined as *P* = *I*^2^*R* (*I* = current, *R* = resistance). As the mention above, for the same applied voltage, a lower number of dip-coatings results in lower electrical heating properties. It was occurred because the number of graphene particles that have electrical heating properties was small. Also, the electrical heating properties were improved as the number of coats increased because it was attributable to the increase in the number of graphene particles.

As the results of electrical heating behavior for confirming the application of fabric electrical elements was indicated in Fig. 1(g). When the 50 V was applied to the coated samples, a steady-state temperature can be reached in less than 10 min and maintained for 30 min. Liu *et al*.^9^ reported the composite fabric prepared in an aqueous dispersion containing 0.080 wt% rGO, a steady-state temperature of 59 °C reached in less than 30 min at 30V. Thus, the electro-conductive para-aramid knit has been found to reach steady-state temperature sooner. And it was confirmed that could be maintained the electrical heating performance for long time used.

**Figure 2:**
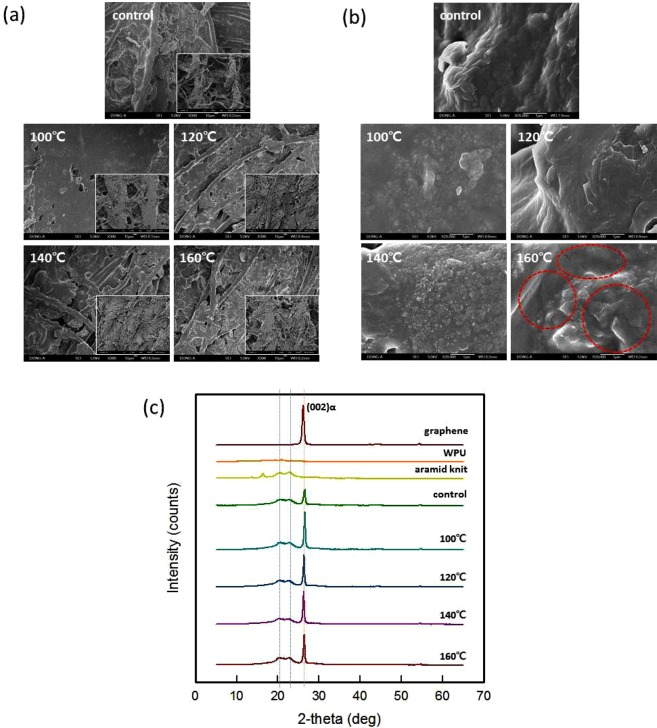
Scanning electron microscopy (SEM) micrograph of the surface of the five-coat knits with different hot-press temperatures (**a**) at 300x magnification, 50x magnification (inserted image), and (**b**) 20,000x magnification and (**c**) XRD patterns.

As shown in Fig. 2(a), the control sample was shown that there was a space between the course and the wale of the para-aramid knit even after coating. Since all the samples have undergone process of drying and curing, it was confirmed that the distilled water remaining in the graphene/WPU coating on the para-aramid knit were evaporated and formed pores. At this process, it was highly porous, which was led to high contact resistance and unsmooth pathways for electron transport^32^. And increased samples density with compression process such as and hot-pressing^27^ and rolling^32^ could be increased the conductivity due to the conductive pathway increases. Therefore, this study was conducted the hot-press process applied at various temperatures to enhance the electrical properties.

SEM images of the hot-pressed samples (Fig. 2(a)), all the hot-pressed samples were shown a smooth surface, but at 100 °C, that were voids between the courses and wales those were not filled. After hot pressing the samples at 120 °C, 140 °C and 160 °C, the voids in the knit were packed with graphene dispersion. In the SEM images at 20,000x magnification(Fig. 2(b)), hot pressed samples at 160 °C had some cracks on the surface. It was confirmed that to be the effect of WPU which was used as a polymer. At 100 °C, the WPU had slightly melted and fluidity. However, at 120 °C and above, it is confirmed that the void area was packed with graphene/WPU composite because the fluidity increased when the WPU was melted and pressure was applied. And the thermal stability of the WPU was lowered at 160 °C, thus some cracks could be seen in hot pressed samples at 160 °C(Fig. S2).

As the results of XRD pattern of the electro-conductive textile by different hot-press temperatures(Fig. 2(c)), the diffraction patterns of the knits presented three sharp peaks at 2θ = 16.4°, 20.5°, and 22.7°, and WPU was shown a peak at 2θ = 20.8°. In the coated knit, the dominant peak was around 26.2°, which was ascribed to graphene. The XRD patterns of the coated knits exhibited more defined graphene peaks than that of the control sample. Chen *et al*.^33^ reported that the intensity of the diffraction signal originating from carbon-based nanofiller increases with respect to the polymer-based crystal diffraction peaks. This results from the alignment of graphite flake fillers inside a polymer matrix. Thus, the graphene inside the WPU matrix could be re-oriented after hot-pressing and established a conjugated graphene network in the graphene/WPU composite. When hot-pressing at 160 °C, there was slightly lower intensity, which could be explained by the morphology in the SEM images. It is due to the samples hot-pressed at 160 °C showed cracks on the surface, which act as defects in the crystal properties and electrical properties.

**Figure 3:**
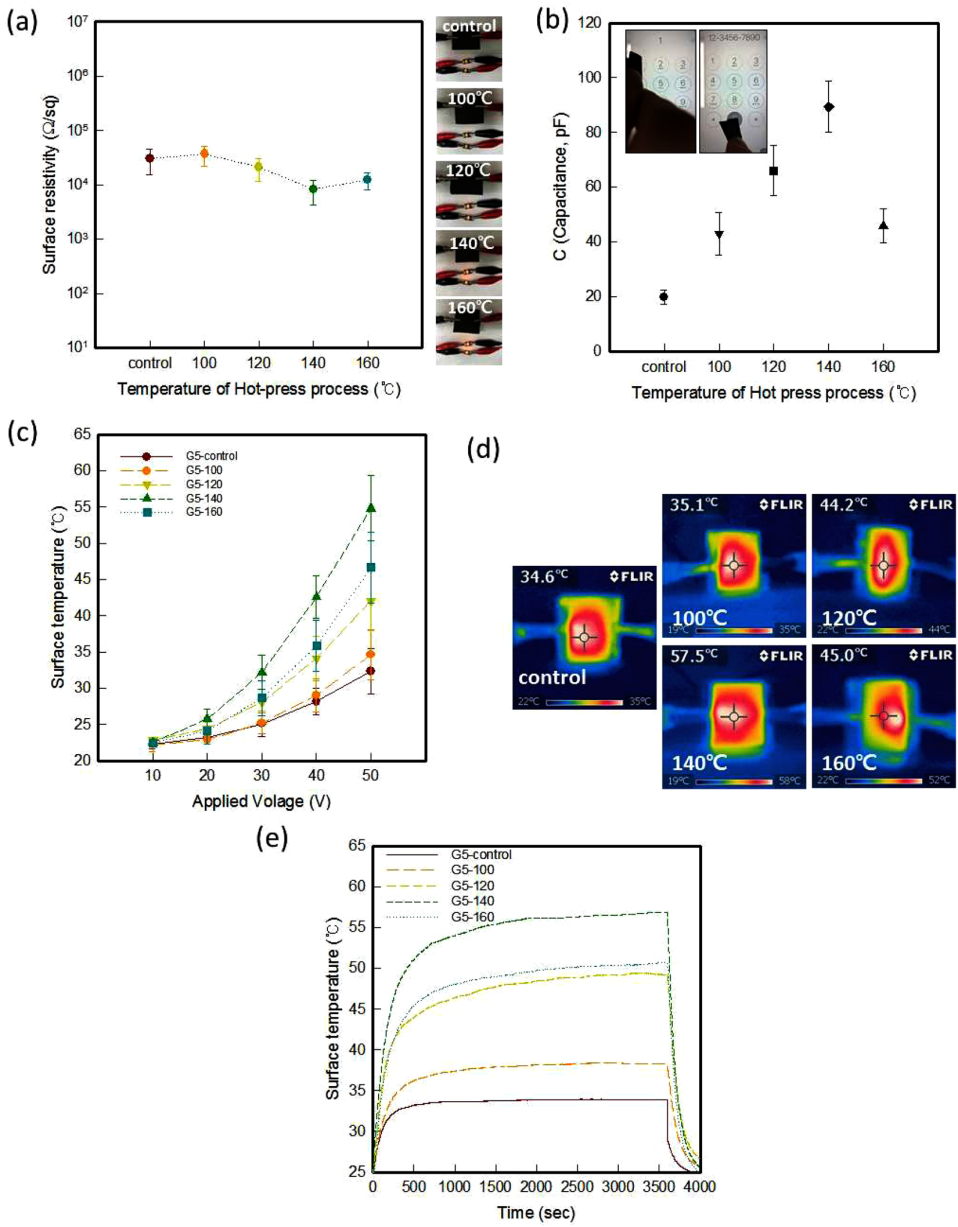
(**a**) Surface resistivity of para-aramid knit dip-coated with graphene/WPU composite and the electrical conductivity for the five-coat samples for different hot press temperatures (insert images). (**b**) Capacitance of the coated knit hot-pressed at 140 °C on a touchscreen (insert image). (**c**) Surface temperature of coated knit with different hot press temperatures and applied voltages. (**d**) Thermal image of five-coat knit with 50 V applied and (**e**) Electrical heating behavior of samples by different hot-press temperature with applied 50 V.

The average surface resistivity of the samples hot-pressed at 100 °C, 120 °C, 140 °C, and 160 °C were 6.3 × 10^5^ Ω/sq, 2.5 × 10^4^ Ω/sq, 1.4 × 10^4^ Ω/sq, and 2.1 × 10^4^ Ω/sq, respectively (Fig. 3(a)). The surface resistivity of the samples hot-pressed at 100 °C slightly increased, those at 120 °C and 140 °C were decreased, and that at 160 °C was increased. This could also be explained the morphology in the SEM images and XRD pattern.

The hot-press process was generally used to increase the density of a sample^27^. The samples hot-pressed at 100 °C had less conductive paths because the graphene/WPU composite was not packed in the entire sample. However, the hot-pressed samples at 120 °C and 140 °C were filled with the composite, so more conductive network was generated, and the electrical properties were improved. The hot-pressed samples at 160 °C showed defects in the formation of the conductive paths due to cracks appearing on the surface. Crack formation caused reductions in the electrical conduction and eventual degradation^27^.

When increasing the hot-press temperature from the control to 140 °C, the capacitance increased from 19.8 to 89.4 pF (Fig. 3(b)). As mentioned, the measurement conditions were the same, and the thickness decreased by 50% after the hot-press process according to the formula *C* = *ε* × (*A*/*d*), where ε is the dielectric constant of the dielectric between the electrodes, *A* is the area of the electrodes, and *d* is the distance between the electrodes. This was used for determining the capacitance^34^. An amount of charge can be accumulated in the coated hot-pressed knits. The capacitance of the sample hot-pressed at 140 °C was the largest, and it decreased for the sample hot-pressed at 160 °C. Thus, the sample of hot-pressed at 140 °C was more easily to touch screen than control sample (see Supplementary Video Demonstration). This tendency was also shown in the electric heating properties (Fig. 3(c) and Fig. 3(d)). The hot-pressed samples showed higher surface temperature than the control samples. When we applied 50 V to the sample hot-pressed at 100 °C, it showed a similar surface temperature of about 34.7 ± 3.4 °C. The surface temperature of samples hot-pressed at 120 °C and 140 °C increased to 42.1 ± 4.1 °C and 54.8 ± 4.5 °C, respectively. The surface temperature of the sample hot-pressed at 160 °C decreased to 46.7 ± 4.8 °C. As mentioned for the electrical properties, cracking occurred at the surface of the hot-pressed sample at 160 °C^26^, which influenced the capacitance and electrical heating properties. And the results of electrical behavior of samples by different hot-pressed sample could be reached the state-steady temperature less than 20 min and maintained 60 min other variation when applied 50V (Fig. 1(g)). It was confirmed that the electrical heating performance of the electro-conductive textile could be long-lasting.

**Figure 4:**
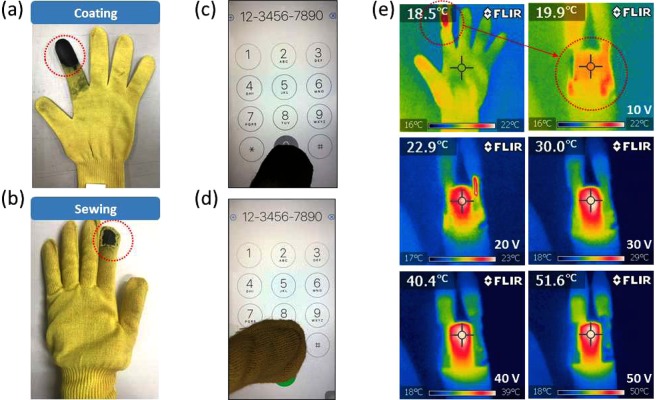
Digital photograph of a graphene/WPU dip-coated glove obtained by (**a**) direct coating and (**b**) sewing. The coating on the glove can be detected well by the touchscreen with both the (**c**) direct coating and (**d**) sewing methods. (**e**) Thermal image of the gloves with voltage applied.

The electro-conductive coating can be applied to a para-aramid glove by directly dip-coating it or sewing dipped fabric onto it (Fig. 4(a,b)). Figure 4(a) shows a glove that was dip-coated on the index finger fifth times and hot-pressed at 140 °C and 3.5 MPa for 3 min (the optimal conditions). Coated fabric was also applied by sewing, as illustrated in Fig. 4(b). Figure 4(c,d) show that both of these methods can be used to make touchscreen gloves for a mobile phone (see Supplementary Video Demonstration).

The surface temperature of the electro-conductive glove is shown in a IR thermal image in Fig. 4(e). When a voltage of 10 to 50 V was applied for 3 minutes at 10-V intervals, the surface temperatures were 19.9 °C, 22.9 °C, 30.0 °C, 40.4 °C, and 51.6 °C, respectively, which was similar to the five-coat sample that was hot-pressed at 140 °C. The dip coating method can thus be used to manufacture knit fabric and a glove that exhibits electric and heating properties.

## Conclusions

In this study, we fabricated elecro-conductive textile using graphene/WPU composite with para-aramid knit fabric by simple dip-coating method, and developed a fabric heating element which could be protected the human body temperature in functional clothing such as working and protective clothing, winter leisure clothing, etc. The electro-conductive para-aramid knit fabrics were prepared by two conditions. The first was prepared according to the number of dip coating cycles from one to five and the second was prepared by the hot-press temperatures using five-coat samples having the best electrical, and specifically electric heating performance. As the number of dip-coating cycles increased from one to five, the electrical and electrical heating performance was improved, and these properties of five-coat samples were more increased after hot-press process due to the improvement of the conductive path as the distance between the graphene grains nears the hot-press process. The best performance was indicated at five-coat sample with hot-pressed at 140 °C, with a surface resistivity and capacitance of 7.5 × 10^4^ Ω/sq and 89.4 pF, respectively. When a voltage of 50 V was applied, the surface temperature reached 54.8 °C, and the steady-state temperature of samples were reached less than 20 min, and it was maintained for 60 min with no variation. Thus, the optimum conditions were hot-pressing at 140 °C and five coats, which were applied to a para-aramid glove using direct dip-coating and sewing. The touchscreen and electric heating properties were the same as the coated fabric sample. The electro-conductive para-aramid knit is expected to be applied to protective and functional clothing requiring high durability.

## Methods

**Figure 5:**
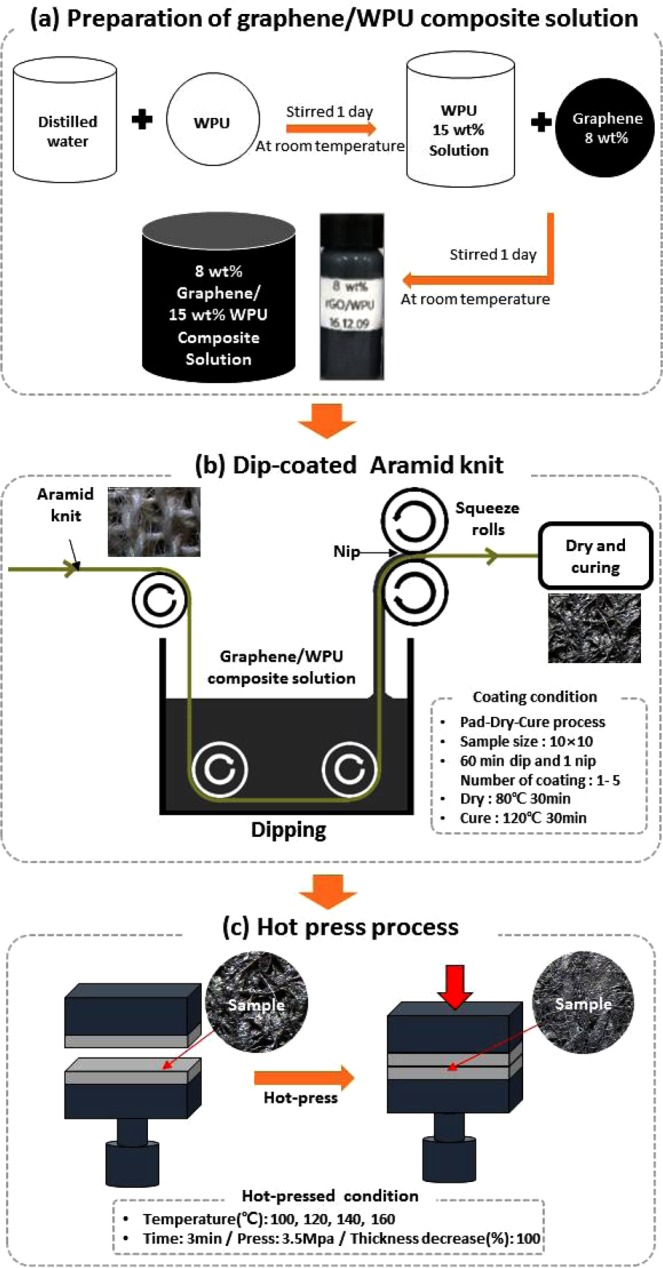
Illustration of the fabrication process for graphene/WPU dip-coated on para-aramid knit, which consists of three steps: (**a**) Preparation of graphene/WPU composite solution, (**b**) Dip-coating para-aramid knit with various coating cycles, and (**c**) Hot-pressing with different temperatures.

Graphene (Carbon Nano Technology Co. Ltd., Korea) was prepared by a proprietary chemical exfoliation method. The graphene has 3–10 layers, lengths of 5–10 mm, and thicknesses of 3–6 nm. Waterborne polyurethane (WPU, STANL HOLDINGS B.V., Netherlands) was used, and the solid content was 50%. The solvent used was distilled water. Para-aramid knit (0.19 g/cm^2^, thickness: 0.97 ± 0.01 mm, Mirae advanced material Co. Ltd., Korea) was used as the substrate fabric. Figure 5 illustrates the fabrication process for the electro-conductive para-aramid knit. To obtain the graphene/WPU composite solution, 15 wt% WPU and 8 wt% graphene were dissolved by stirring. In our previous study^10^, graphene/WPU composite solution and film was fabricated with various graphene contents as 0, 2, 4, 8, and 16 wt%. The aggregation of graphene particles was indicated from 8 wt% graphene/WPU composite film due to the pristine graphene tends to form irreversible agglomerates through van der waals interaction^35^. And the electrical property was indicated from 8 wt% graphene/WPU composite films. And the electrical property was improved 16 wt% graphene/WPU composite film but it was brittle and crushed after the annealing treatment. Thus, the 8 wt% graphene/WPU composite was selected due to the better formability with the electrical properties. The knits were prepared by dip-coating with the graphene/WPU composite solution. For the first dip-coating cycle, the knit was dipped in the solution for 60 min to reach an average wet pickup of 100% and then dried for 30 min at 80 °C in a drying oven (WOF-155, Korea). Subsequent coatings were applied using the same procedure up to five times. Curing was carried out at 120 °C for 30 min. The thickness of the five-coat sample was 1.11 ± 0.05 mm. Afterwards, the five-coat samples were hot pressed at 3.5 bar (Carver Laboratory Press Model 3912, Carver Inc., U.S.A) for 3 minutes at temperatures of 100 °C, 120 °C, 140 °C, and 160 °C. The range of hot-press temperatures was set by thermogravimetric analysis, as shown in Fig. S2. The thickness of the five-coat sample decreased by 50% (0.57 ± 0.01 mm). The hot-press process was used because it has the potential to produce denser materials. Teflon films were used during the hot-pressing procedure to prevent sticking of the nanocomposites on the platen of the press. The hot-pressed samples were cooled down to room temperature for 24 hours and then stored in a desiccator before characterization.

To investigate the morphology of the coated knits, the samples were measured by fabric image analysis microscopy (NT 100, Nextec, Korea) at 6.5x magnification and FE-SEM (JSM-6700F, Jeol, Japan) at 300x and 20,000x magnification. X-ray diffraction (XRD) spectra of the knits were analyzed using an X-ray diffractometer (Ultina IV, Rigaku CO., Japan) using Ni-filtered CuKα radiation. To measure the electrical properties, the surface resistivity was measured by a multimeter (ST850A, Saehan Tester. Co., Korea) based on a modified version of the AATCC-76 method. Two parallel conductive probes were placed in contact with both edges of the samples. The sample size was 25 mm × 25 mm. The surface resistivity *R*_*s*_ is expressed in ohms per square and was calculated as follows: *R*_*s*_ (Ω/sq) = (*W*/*D*) × *R*’, where *R*’ is the resistance measured by the multimeter, *W* is the width of the sample, and *D* is the distance between the two electrodes.

The capacitance of the coated samples was studied using a HIOKI-IM 3570 high-precision impedance analyzer at a frequency of 1 kHz and a voltage of 1 V at room temperature. Samples with the same thickness were measured using two electrodes at the top and bottom with diameters of 2.5 cm. The electrical heating properties were measured with a DC power supply (CPS-2450B, CHUNGPAEMT, Korea). The sample size was 25 mm × 25 mm, and copper tapes were attached to both edges of the sample to connect to the power source via alligator clips. The aim of our research was to fabricate a fabric heating element that exhibits 50 °C. This study was presented over 50 °C when 50 V was applied, thus the applied voltage range was set from 10 to 50 V (DC). A thermal imaging camera (FLIR i5, FLIR Systems INC., USA) was used for measuring the surface temperature when applying the voltages. Used surface temperature was the position of middle of the samples. Five samples were measured and mean value was used. To measure of electrical heating behavior of samples, temperature data logger (TR-71wf, T&D corp., Korea) and temperature sensor (TR-0206, T&D corp., Korea) were used. Samples are applied 50V, and for 1 hour and the temperature values was measured until the temperature reached equilibrium state. Five samples were tested and the average was obtained and then the value was used. And the deviation was from calculated from five samples.

## Supplementary information

Supplementary video_Figure. 1(h)

Supplementary video_Figure. 3(b)

Supplementary video_Figure. 4(c)

Supplementary video_Figure. 4(d)

Supplementary video_Figure. S1(d)

Supplementary information
