# Supplementary material for: Electrical Heating Performance of Electro-Conductive Para-aramid Knit Manufactured by Dip-Coating in a Graphene/Waterborne Polyurethane Composite
Source: Sci Rep. 2019 Feb 6;9:1511. doi: 10.1038/s41598-018-37455-0 (PMC6365530; doi:10.1038/s41598-018-37455-0)
Supplement: Supplementary file 6 — Supplementary information [file 41598_2018_37455_MOESM6_ESM.docx]

**Supplementary Information**

**Electrical Heating Performance of Electro-Conductive Para-aramid Knit Manufactured by Dip-Coating in a Graphene/Waterborne Polyurethane Composite**

Hyelim Kim^1,3^, Sunhee Lee^2^*, and Hanseong Kim^3^*

^1^Research Institute of Convergence Design, Dong-A University, Busan, 49315, Rep. of Korea. ^2^ Dept. Fashion Design, Dong-A University, Busan, 49315, Rep. of Korea. ^3^ Dept. Organic Material Science and Engineering, Pusan National University, Busan, 46241, Rep. of Korea. Correspondence an requests for materials should be addressed to S.H.L. (e-mail: shlee014@dau.ac.kr) or H.S.K.(hanseongkim@pusan.ac.kr)


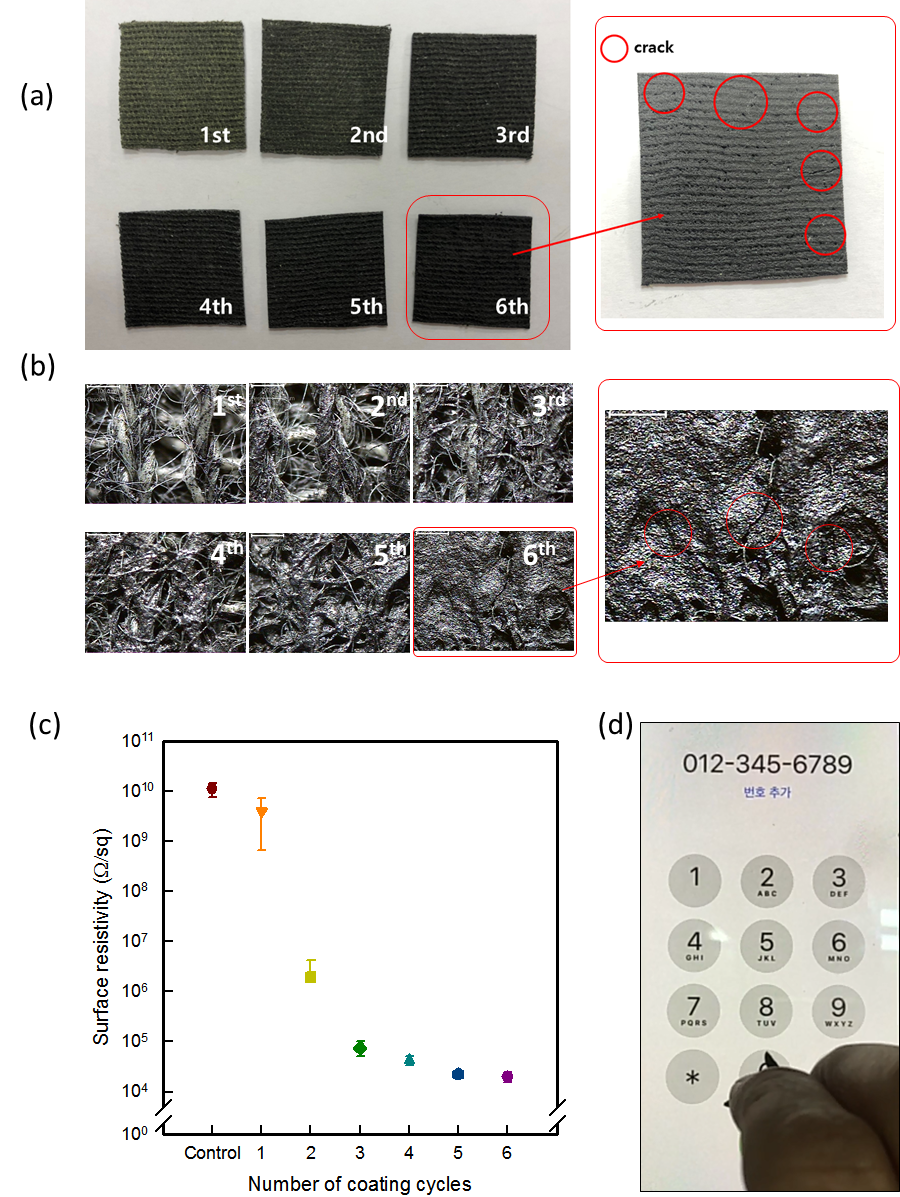


**Figure S1. (a) Digital image, (b) surface morphology and (c) surface resistivity of dip-coated sample up to six-coat with graphene/WPU composite solution (inset image shows using the six-coat samples to touch a screen) (d)** **Using the six-coat samples to touch a screen.**

We did dip-coating up to six times. However, some cracks were formed on the surface after the dip-dry-cure process for six-coat samples as seen in Fig. S1 (a) and (b). It could be an obstacle to the connection of the conductive path. As seen in Fig. S1 (c), the surface resistivity of the six-coat indicated 2.0×10^4^ ± 4.5×10^3^ Ω/sq, it was similar value to five-coat samples. The six-coat sample also could be used to touch a screen (Fig. S1(d) and see supplementary video demonstration). As the work process becomes longer in the mass production, the time and cost become wasted. Thus, in this study, the samples with five-coat is more suitable than the six-coat sample.

**Figure S2. Thermogravimetric analysis of the WPU film and 8 wt% graphene/WPU composite film.**

We performed TGA of the WPU film and 8 wt% graphene/WPU composite film. The results are reported in Figure S2. The weight loss is measured as a function of the temperature, no significant weight loss is recorded until 250°C. Based on that, the hot-pressing temperature range set at 100°C to 160°C, thus preventing thermal degradation, ensuring WPU melting, and confirm the characteristics at different hot-pressing temperatures. Graphene are not degraded during the measurements and maintain their initial mass at 600°C.
